# Detection and isolation of airborne SARS‐CoV‐2 in a hospital setting

**DOI:** 10.1111/ina.13023

**Published:** 2022-03-27

**Authors:** Nuno Rufino de Sousa, Laura Steponaviciute, Lucille Margerie, Karolina Nissen, Midori Kjellin, Björn Reinius, Erik Salaneck, Klas I. Udekwu, Antonio Gigliotti Rothfuchs

**Affiliations:** ^1^ Department of Microbiology, Tumor and Cell Biology (MTC) Karolinska Institutet Stockholm Sweden; ^2^ Department of Medical Sciences Infectious Diseases Uppsala University University Hospital Uppsala Uppsala Sweden; ^3^ Department of Medical Biochemistry and Biophysics (MBB) Karolinska Institutet Stockholm Sweden; ^4^ Department of Aquatic Sciences and Assessment Swedish University of Agricultural Sciences Uppsala Sweden

**Keywords:** environmental sampling, health care, infectious aerosols, pathogen detection, SARS‐CoV‐2, transmission

## Abstract

Transmission mechanisms for severe acute respiratory syndrome coronavirus 2 (SARS‐CoV‐2) are incompletely understood. In particular, aerosol transmission remains unclear, with viral detection in air and demonstration of its infection potential being actively investigated. To this end, we employed a novel electrostatic collector to sample air from rooms occupied by COVID‐19 patients in a major Swedish hospital. Electrostatic air sampling in conjunction with extraction‐free, reverse‐transcriptase polymerase chain reaction (hid‐RT‐PCR) enabled detection of SARS‐CoV‐2 in air from patient rooms (9/22; 41%) and adjoining anterooms (10/22; 45%). Detection with hid‐RT‐PCR was concomitant with viral RNA presence on the surface of exhaust ventilation channels in patients and anterooms more than 2 m from the COVID‐19 patient. Importantly, it was possible to detect active SARS‐CoV‐2 particles from room air, with a total of 496 plaque‐forming units (PFUs) being isolated, establishing the presence of infectious, airborne SARS‐CoV‐2 in rooms occupied by COVID‐19 patients. Our results support circulation of SARS‐CoV‐2 via aerosols and urge the revision of existing infection control frameworks to include airborne transmission.

**Practical implications**

- Support for airborne route of viral transmission by the demonstration of active SARS‐CoV‐2 in air from COVID‐19 patient rooms and adjoining indoor environments.
- Support for aerosol transmission‐based mitigating measures against SARS‐CoV‐2 in healthcare settings.

## 1 INTRODUCTION

SARS‐CoV‐2 is the causative agent of coronavirus disease 2019 (COVID‐19), which has since March 23, 2022 claimed more than 6 million deaths worldwide.^1^ At the onset of the COVID‐19 pandemic, medical treatments such as vaccination were unavailable. Infection prevention and control measures targeted instead the development of diagnostics and implementation of physical distancing, local and countrywide lockdowns, and disinfection protocols. Much research has since been dedicated to unfolding the routes of transmission of the virus, including the contribution of droplets, aerosols, and fomites. However, early reports emphasized droplets and fomites in transmission.^2^, ^3^ Physical‐distancing guidelines to mitigate the spread of SARS‐CoV‐2 have advocated often vague and country‐variable “safe physical distancing” in workplaces,^4^ keeping when possible, at least 6 feet (~1.8 meters) between people in healthcare facilities^5^ and at least 1 meter distancing in school settings.^6^ Reports on super‐spreading events^7^, ^8^, ^9^ and detection of SARS‐CoV‐2 RNA in hospital air^10^, ^11^, ^12^, ^13^, ^14^, ^15^ have suggested, however, that the virus may also spread through aerosols. These accounts are accumulating but are each based on limited datasets. They have also been countered by reports of negative detection of the virus in air,^16^, ^17^, ^18^ and systematic reviews rejecting airborne transmission of the virus.^19^, ^20^

Today, the CDC and the WHO acknowledge not only the airborne route of transmission but also that the claim is not well‐established and experimental support limited.^5^, ^21^, ^22^ Confirmation of active SARS‐CoV‐2 in air remains to be thoroughly demonstrated to dispel further speculation; more studies and robust datasets are needed to establish aerosols as central to transmission of SARS‐CoV‐2 in the environment. A key step to establish aerosol transmission is to demonstrate the virus in air and on surfaces that cannot be explained by virus droplet deposition at that site. Such demonstration requires the combination of tactical microbiological air sampling with detection methods needed to establish the presence of airborne SARS‐CoV‐2. In this regard, electrostatic precipitation is emerging as a simple and efficient way to collect bioaerosols.^23^, ^24^, ^25^, ^26^ Herein, we used an electrostatic air sampler developed in our laboratory^27^ in conjunction with an extraction‐free RT‐PCR for SARS‐CoV‐2^28^ and standard viral PFU assays. We investigated the presence of airborne SARS‐CoV‐2 in COVID‐19 patient rooms and adjoining indoor environments in a Swedish healthcare setting. Airborne investigation was performed alongside detection of the virus on out‐of‐reach and high‐contact surfaces in the same space.

## 2 MATERIALS AND METHODS

### 2.1 Environmental sampling

Sample collection was performed in the infectious disease ward of Uppsala University Hospital, Uppsala Sweden, on four separate occasions (December 10, 2020, January 19, 2021, January 21, 2021, and February 23, 2021). Room air was collected using the Tuberculosis Hotspot detector (THOR) electrostatic air sampler.^27^ THOR uses electrostatics in an open‐air environment. It ionizes airborne particles and accelerates them through an electrostatic field toward a stainless‐steel collector on its center. Rooms housing patients with clinically PCR‐confirmed COVID‐19 and the adjoining anteroom to each patient room were simultaneously sampled on separate THOR devices for 15 min. After sampling, used collector pieces were replaced with new ones and air sampling repeated for another 15 min. THOR was placed on a tripod at 1 m from the ground and at least 2 m from the patient. Collector pieces were transferred into 0.5 ml PBS containing 0.05% Tween‐80 (PBS‐T)(Sigma) and vortexed for 1 min.^27^ THOR devices were disinfected with 70% EtOH before moving to a new sampling location.

Patient rooms had on average a floor area of 25 m^2^ in size and were 65 m^3^ in volume (Table 1). Two patients could be housed in these rooms at once. Surface samples from the room's air exhaust ventilation, the railing of the patient's bed and the floor next to the bed were collected in patient rooms prior to air sampling. The surface of the air exhaust ventilation was also swabbed in the anteroom. Surface samples were collected using MS Mini DNA/RNA buccal swabs (Isohelix, Cell Projects). Two swabs were used simultaneously on the same surface area, stored in 3 ml eNat^®^ preservation buffer (COPAN) and processed separately. In rooms with two occupants, each bed rail was sampled with one individual swab. The total surface area sampled at each location was ~90 cm^2^ for air exhaust vents, ~100 cm^2^ for bed rails and ~625 cm^2^ for the floor. Indoor room air temperature, relative humidity, and CO_2_ levels were measured using a pSENSE II environmental logger (Senseair AB). An illustration of THOR, the approximate location of THOR, approximate location of the environmental logger, and of surface sampling in patient and adjoining anterooms are provided (Figure S1). The full layout of the ward has been previously reported.^29^

**TABLE 1:** Parameters of the sampled patient rooms and anterooms^a^

	Patient room^b^	Anteroom
Area (m^2)^ (IQR)	25.02 (19.75–25.60)	6.02 (5.80–6.120)
Volume (m^3^) (IQR)	65.58 (51.52–67.50)	14.45 (13.89–14.63)
Air‐changes per hour (IQR)	2.4 (2.2–2.65)	ND
Air temperature (°C) (IQR)	23.10 (22.70–23.40)	23.10 (22.70–23.80)
Relative humidity (%) (IQR)	23.70 (21.30–31.20)	26.20 (24.50–28.90)
CO_2_ concentration (ppm) (IQR)	660 (630–878)	782 (654–878)

Note

Patient and anteroom metadata (area and volume) and environmental parameters (air‐changes‐per‐hour, air temperature, relative humidity, and CO_2_ concentration). Percentages (%) and interquartile range (IQR) given.

Abbreviation: ND, not determined.

^a^ Median.

^b^ Including bathroom.

Ten different patient rooms with adjoining anterooms were sampled in the above way. One patient room and adjoining anteroom were sampled twice but with more than 3 weeks interval between sampling. Thus, we considered our samples to include a total of 11 groups of rooms. In addition, control air sampling was done in 1 physician office and 1 physician meeting room, both located just outside the ward and empty at the time of sampling. Air sampling was also performed in 2 patient rooms and their adjoining anterooms which had been cleaned as per standard ward routines and unoccupied for at least 2 days.

### 2.2 Ethics statement

The study was conducted according to good clinical and scientific practices and following the ethical principles of the Declaration of Helsinki. Approval for accessing patient information and patient samples was granted from the Swedish Ethical Review Authority under the study protocols DNR 2020–01787 with amendment DNR 2021–00072. Patient nasopharynx samples taken for clinical diagnosis of COVID‐19 were retrospectively obtained from the Uppsala Biobank.

### 2.3 Extraction‐free RT‐PCR on heat‐inactivated samples (hid‐RT‐PCR)

Hid‐RT‐PCR was performed on heat‐inactivated aliquots of all samples as previously described,^28^ with a few modifications. Primer and probe sequences used are the same as in the original publication.^28^ Briefly, after heat inactivation at 95°C for 5 min, 12 μl of the inactivated sample was added to 48 μl RT‐PCR mastermix consisting of 15 μl of one‐step TaqPath RT‐qPCR master mix (Thermo, A15299), 4.5 μl of primer‐probe mix, 1.8 μl of 10% Tween‐20 solution, and nuclease‐free water to achieve a final reaction volume of 60 μl. The N1 primers and probe were used at a concentration of 500 nM and 125 nM, respectively. The thermal cycling steps were 25°C for 2 min, 50°C for 15 min, 95°C for 2 min, and 45 cycles of 95°C for 3 s and 56°C for 30 s. In RT‐qPCR run, nuclease‐free water was used as negative control and heat‐inactivated *in vitro* expanded SARS‐CoV‐2^28^, ^30^ was used as positive control. The limit of detection for this reaction is 2–20 genome copies/µl.

### 2.4 RNA‐extraction‐based RT‐qPCR

Total nucleic acid was extracted from air samples collected in PBS‐T or from swab samples collected in eNat^®^ preservation buffer (COPAN) using the Zymo Quick‐DNA/RNA^TM^ Viral MagBead kit (Zymo Research) following the recommended instructions and eluted in 50 µl of nuclease‐free water. RT‐qPCR was performed according to manufacturer's recommendations using the Quick SARS‐CoV‐2 rRT‐PCR Kit (Zymo Research). Briefly, eluted air‐ or swab‐derived total nucleic acid was added to Master‐mixes containing either primers targeting N1, N2, and N3 regions of viral nucleocapsid gene tagged with the HEX^™^ fluorophore, or the human RNaseP gene tagged with a Quasar^®^ 670 fluorophore. Two serially diluted standards (provided in the kit) were used. The thermal cycling steps were 55°C for 15 min, 95°C for 10 min, and 45 cycles of 95°C for 5 s and 57°C for 30 s. The manufacturer reports 15 genome copies/reaction as the limit of detection of this reaction.

### 2.5 Isolation and quantification of SARS‐CoV‐2

Quantification of Plaque‐forming units (PFUs) was performed on 90% confluent Vero E6 cells (ATCC‐CRL‐1586), using 6‐well tissue‐culture plates. Briefly, environmental samples were serially diluted with DMEM and added to Vero E6 cells for 1 h at 37°C followed by removal of the inoculum media and two washes with PBS. Overlay medium consisting of 2:3 mix of 3% carboxymethyl cellulose and DMEM was added, and the plates were incubated at 37°C for 3 days. Plates were then assessed under the microscope for cytopathic effects in line with viral‐induced plaque formation and marked. For the confirmation of SARS‐CoV‐2 in PFUs from environmental samples, a 50 µl aliquot was obtained directly from the plaque by pipette aspiration of the semi‐solid media using a wide bore 200 µl pipette tip, 50 µl of PBS were added to further solubilize the media and make it amiable to further processing. The presence of SARS‐CoV‐2 RNA in the plaque was determined by hid‐RT‐PCR.

### 2.6 Data analyses

Graphical representations of data and statistical testing were performed using GraphPad Prism 9.3.1 (GraphPhad Software Inc).

## 3 RESULTS

### 3.1 Detection and distribution of SARS‐CoV‐2 RNA in air and on surfaces

**FIGURE 1:**
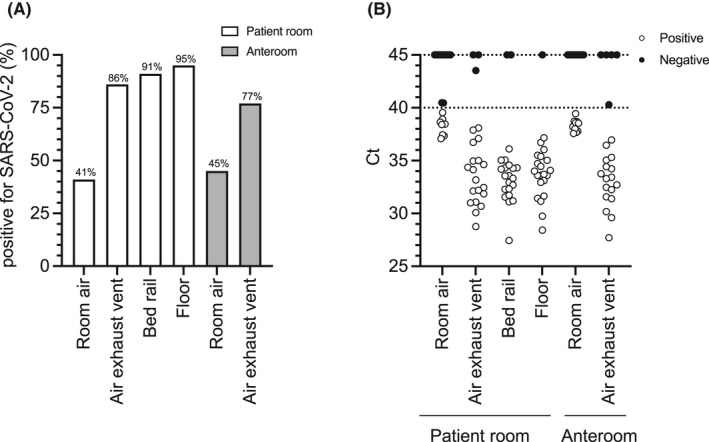
Air and surface sampling from patient rooms and adjoining anterooms. Air and surface samples were collected from patient rooms and adjoining anterooms and tested for the presence of SARS‐CoV‐2 by hid‐RT‐PCR. (A) Percentage of positive air and surface samples across different sampling locations. (B) Ct distribution of the samples collected in different sampling locations. Circles represent individual samples. Average Ct for patient room locations are 38.27 (room air), 33–49 (air exhaust vent), 33.08 (bed rail), and 33.62 (floor). Average Ct for anteroom locations are 38.32 (room air) and 32.99 (air exhaust vent)

Environmental sampling was performed in the infectious disease ward at the 940 bed, university teaching hospital, Uppsala University Hospital, Uppsala Sweden. Air and surface samples were collected from 11 rooms occupied by 15 patients with confirmed COVID‐19. One patient was asymptomatic, the remaining patients were symptomatic and in their second week of respiratory symptoms (Table 2). A quarter of the individuals were also experiencing gastrointestinal symptoms (Table 2). Eleven sets of samples were collected from each patient room and its adjoining anteroom. Both air and surface samples from this collection were positive for SARS‐CoV‐2 RNA as determined by hid‐RT‐PCR (Figure 1A,B). In patient rooms, the bed rail (20/22; 91%) and floor (21/22; 95%) were highly contaminated with SARS‐CoV‐2 RNA (Figure 1A). Contamination was also prominent in samples collected from the surface of ceiling air exhaust vents in patient rooms (19/22; 86%) and in the adjoining anterooms (17/22; 77%) (Figure 1A).

**TABLE 2:** Characteristics of room occupants at the time of environmental sampling

Median age (IQR)	67 (58–74)
Male sex (%)	5 (33)
Median days since onset (IQR)	11.5 (7–14)^a^
Median days at the ward (IQR)	3 (1–4)
Symptomatic (%)	14 (93)
Respiratory symptoms (%)	14 (93)
Gastrointestinal symptoms (%)	4 (27)

Note

Baseline characteristics of COVID‐19 patients occupying rooms at the time of sampling. Percentages (%) and interquartile range (IQR) given.

^a^ One patient was asymptomatic and thus excluded.

Detection of SARS‐CoV‐2 RNA in air was similar between patient rooms (9/22; 41%) and adjoining anterooms (10/22; 45%) (Figure 1A). Average Ct values for positive air samples were 38.27 and 38.32 for patient and anterooms, respectively (Figure 1B). Average Ct values for the bed rail (33.08), patient room floor (33.62), air exhaust vent in the patient room (33.49), and air exhaust vent in the anteroom (32.99) were slightly lower than for positive air samples. Overall, molecular detection indicated a low but consistent viral burden in air and contaminated surfaces in these indoor environments. Importantly, SARS‐CoV‐2 RNA was not detected in control environments (physician office and staff meeting room). Detection was also negative in two patients and adjoining anterooms cleaned after patient discharge and kept unoccupied for at least 2 days.

**FIGURE 2:**
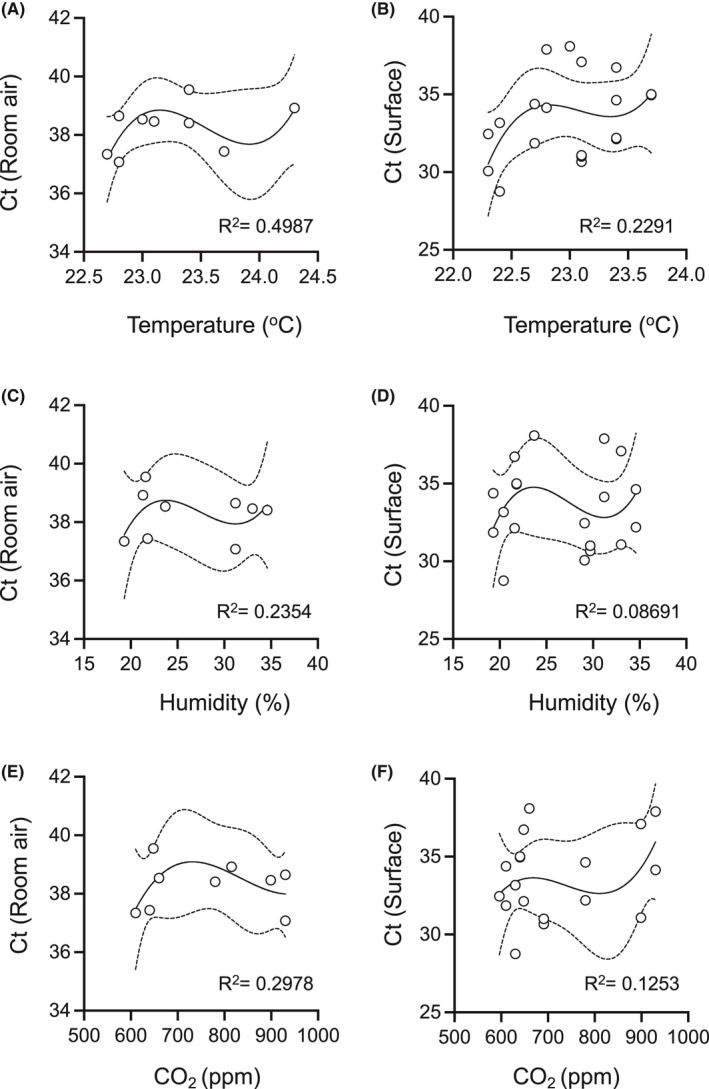
Environmental parameters do not correlate with detection of SARS‐CoV‐2 in air. Environmental parameters were recorded in patient rooms before air sampling. Ct values obtained from air samples and the air exhaust vent surface swabs by hid‐RT‐PCR are plotted against room air temperature (A and B), relative humidity (C and D), and CO_2_ concentration (E and F). Continuous line represents the best fit regression curve (3rd order polynomial) and dashed lines the 95% confidence intervals. No correlations were observed for the given parameters. *R* ^2^ values for each curve shown

To investigate the impact of basic room atmospheric parameters on virus detection, we attempted to match air temperature, relative humidity, and CO_2_ concentration against virus RNA Ct values from the patient room air and the surface of the air exhaust vent. Correlation of given environmental parameters with the detection of virus RNA was, however, not observed (Figure 2). Moreover, viral RNA Ct values in air or on investigated surfaces in the patient room did not correlate with Ct from patient upper respiratory swabs (Figure S2).

### 3.2 Detection and distribution of active SARS‐CoV‐2 in environmental samples

Despite a large return of positive air samples for SARS‐CoV‐2 RNA, it remained to be determined whether active virus particles could be recovered from the same air‐sample material. Thus, we attempted to isolate SARS‐CoV‐2 from samples collected from patient and anterooms. Air samples were inoculated onto a monolayer of Vero E6 cells in *in vitro* culture and surprisingly plaque‐forming units (PFUs) were detected from a significant number of air samples (Table 3). SARS‐CoV‐2 RNA could subsequently be amplified by hid‐RT‐PCR from approximately one‐third of all detected PFUs (496/1472; 34%). Most of the SARS‐CoV‐2 (+) PFUs were isolated from anterooms (320/496; 65%), where also more SARS‐CoV‐2+ PFUs were observed (Table 3). Active SARS‐CoV‐2 was detected in 3 patient rooms and 8 anterooms across three different sampling days (Table 4). A fraction of the air samples positive by hid‐RT‐PCR yielded PFU‐positive cultures, with the highest prevalence in the anteroom (8/10; 80%) (Table 4). These observations establish the presence of infectious, airborne SARS‐CoV‐2 in rooms occupied by COVID‐19 patients and in the airspace of adjoining anterooms. PFU data were also considered in the context of molecular detection of the virus. In the event of a positive PCR result for virus in room air, the likelihood of also recovering infectious SARS‐CoV‐2 upon culturing was 33% in patient rooms and 80% in anterooms (Table 4). Although this molecular detection does not necessarily predict infectiousness, it does suggest an increased risk of encountering infectious virus in environments that test positive for virus RNA.

**TABLE 3:** Detection of infectious SARS‐CoV‐2 particles in air

	Patient room	Anteroom
Total PFUs	672/1472 (46%)	800/1472 (54%)
Median PFUs (IQR; SD)^‡^	48 (16–64; 32.6)	48 (32–48; 20.9)
SARS‐CoV−2+ PFUs/Total PFUs	176/1472 (12%)	320/1472 (22%)
SARS‐CoV−2+ PFUs	176/496 (35%)	320/496 (65%)
Median SARS‐CoV−2+ PFUs (IQR; SD)*	16 (16–16; 10.7)	32 (16–48; 15.1)
SARS‐CoV−2+ PFUs/ml (IQR; SD)	40 (40–40; 26.7)	80 (40–120; 37.7)

Note

PFUs were isolated on Vero E6 cells from air samples. Total and median PFUs from patient and anterooms are shown. The presence of SARS‐CoV‐2 RNA in individual plaques was confirmed by hid‐RT‐PCR (SARS‐CoV‐2+ PFUs). Total PFUs of SARS‐CoV‐2, median PFUs and median PFUs/ml of SARS‐CoV‐2 are shown. Percentages (%), interquartile range (IQR) and standard deviation (SD) given. ^‡^ *p* = 0.8434. *p = 0.0626 Mann‐Whitney test.

**TABLE 4:** Detection of SARS‐CoV‐2 by PCR and plaque assay

Detection	Patient room	Anteroom	Patient room and adjoining anteroom
hid‐RT‐PCR	9/22 (41%)	10/22 (45%)	6/11 (54%)
PFU recovery	3/9 (33%)	8/10 (80%)	3/6 (50%)

Note

Summary of the detection of SARS‐CoV‐2 in the air in the sampled patient rooms and anterooms by PCR and their respective PFU recovery. Percentages (%) given.

## 4 DISCUSSION

The distinction between droplet‐ and aerosol‐based transmissions can be semantic, as both can effectively transmit pathogens, including respiratory viruses.^31^ Our study confirms the detection of SARS‐CoV‐2 RNA in air as well as on ceiling air exhaust vents from COVID‐19 patient rooms, and in adjoining anterooms in the infectious disease ward of a major Swedish hospital. Importantly, our study is the first to recover PFUs of SARS‐CoV‐2 from air. SARS‐CoV‐2 RNA and active virus particles were detected at more than 2 meters from the patient, the only occupant and productive reservoir of virus in the room. Cumulatively, our results provide support for aerosol transmission of SARS‐CoV‐2 and demonstrate the applicability of targeted sampling approaches that deploy air and surface monitoring of respiratory pathogens as previously applied in subway systems.^32^

Detection of SARS‐CoV‐2 on the patient's bed rail and floor may reflect respiratory droplet deposition. We did not quantify PFUs from these sites. Laboratory experiments show that it takes several hours for SARS‐CoV‐2 to become inactivated on metal and plastic surfaces.^33^ Detecting active SARS‐CoV‐2 on high‐contact surfaces may support fomite transmission of the virus, which remains incompletely understood and experimentally contested by some.^34^, ^35^ On the other hand, recovery of SARS‐CoV‐2 from the surface of air exhaust vents in patient and anterooms, implicates detection that is not only more than 2 meters away from the patient but also at a height not directly accessible through contact (2.7 meters). The latter observation together with the demonstration of SARS‐CoV‐2 in air, including recovery of PFUs from air, supports aerosol transmission of the virus.

A limitation of several prior studies showing virus in air has been the singular reliance of RT‐PCR for virus detection.^10^, ^11^, ^12^, ^15^ Another has been the inability to recover PFUs from RT‐PCR‐positive air samples^13^, ^14^, ^36^, ^37^ or central exhaust filters exchanging air from COVID‐19 wards.^29^ In our study we used hid‐RT‐PCR for molecular detection of virus in air. This method has the advantage of being quick, scalable, reliant on off‐the‐shelf reagents, low‐cost on a per‐reaction basis and safer due to a heat inactivation step prior to sample handling.^28^ Detection of SARS‐CoV‐2 RNA by (extraction‐free) hid‐RT‐PCR returned higher Ct values for air samples compared to regular extraction‐based RT‐qPCR but not for surface samples (Figure S3). Nevertheless, our Ct returns are consistent with prior reports of environmental air sampling for SARS‐CoV‐2 in hospital settings.^13^, ^37^ Our demonstration of SARS‐CoV‐2 by both molecular methods and culture implies that detection of virus RNA in air and on surfaces are indicative of active virus. In lieu of accurate infectious dose estimates, hospital environmental samples with high Ct returns for SARS‐CoV‐2 should not be deemed free of infection potential. Instead, aerosol‐based infection control and prevention measures should be considered also following such observations.

The higher number of PFUs recovered in our study were isolated from anterooms. This is consistent with the design intervention of the rooms in the ward, which were built to generate the lowest relative pressure in the anterooms. This enables the patient rooms to be used both for isolation of patients with airborne infectious diseases as well as for protective isolation of immunocompromised patients. Unfortunately, the air filtration in the investigated patient rooms was well below the recommended 12 air‐changes‐per‐hour. This probably increased our ability to culture SARS‐CoV‐2 from air in the rooms, indicating the importance of air dilution interventions in mitigating transmission of airborne pathogens.^38^ Indeed, the lack of recoverable SARS‐CoV‐2 PFUs from environmental air samples remains an obstacle in establishing airborne transmission of the virus.^19^ Cell cytopathic effects have been observed on Vero E6 cells after co‐culture with hospital air samples.^13^ In that study, air samples originated from a single COVID‐19 patient/patient room and PFU assays were not performed. Air sampling from two domestic rooms with single COVID‐19 occupants also failed to demonstrate PFUs of SARS‐CoV‐2.^39^ Our study is the first to recover of PFUs of SARS‐CoV‐2 from air and in more than one indoor environment. Indeed, we isolated PFUs from a total of 11 different rooms (3 patients and 8 anterooms) across three sampling occasions. PFUs of SARS‐CoV‐2 have been recovered from the air of cages housing infected hamsters.^40^ Our results are also in line with seminal laboratory experiments showing that SARS‐CoV‐2 maintains infection potential in aerosols for several hours.^33^, ^41^

We were surprised to recover active SARS‐CoV‐2 from air. Inactivation of bacteria or viruses is expected during electrostatic air sampling,^42^, ^43^, ^44^ including on our own THOR collector.^27^ Interestingly, a recent air sampling study performed in the same infectious disease unit was unable to retrieve active virus from air using a different electrostatic collector.^36^ Our shorter sampling interval and ability to rapidly immerse sample into buffer may have helped preserve viral activity in our material. We speculate that an outer cuff of viral particles shielded a smaller number of virions in the interior of the aerosol from the inactivation effects of ionization, aerosol stress and/or impaction onto the collector. Indeed, impactors, impingers, or cyclone samplers are more readily used to recover active virus compared to electrostatic samples.^45^ Our laboratory tests show that a high titer of SARS‐CoV‐2 is inactivated by almost 2 orders of magnitude after 15 min of THOR operation (Figure S4). Thus, it is not surprising that the number of PFUs recovered from THOR air sampling was low. Given that the sampler used in this study was not adapted for recovery of active virus particles from air, the true viral load may be substantially higher than the values reported.

We did not observe a correlation between symptoms, disease onset or Ct returns from patient upper respiratory swabs and the detection of SARS‐CoV‐2 in air or surfaces. An early study also failed to couple symptoms with virus shedding as measured by contamination of air and surfaces in the infected patient's vicinity.^12^ That said, our patients were entering their second week of disease, and SARS‐CoV‐2 titers in the upper respiratory tract tend to peak in the first week of disease.^46^

In summary, our study substantially contributes to the growing body of experimental and scientific arguments advocating aerosols as critical component of the transmission of SARS‐CoV‐2. Aerosols can remain airborne for extended periods of time and travel long distances with air currents unless they are actively removed by filtration. Acknowledging the aerosol route of transmission for SARS‐CoV‐2 has consequences for infection control and prevention measures in healthcare settings and the built environment that merit immediate attention.

## CONFLICT OF INTEREST

The authors declare no conflicts of interest.

## AUTHOR CONTRIBUTION

NRS, LS, LM, BR, and KN performed experiments. MK provided materials. NRS, BR, KN, ES, KIU, and AGR analyzed the data and prepared figures. KIU and AGR supervised the study. NRS, ES, KIU, and AGR wrote the manuscript. All authors participated in manuscript editing and approved the manuscript.

## Supporting information

Fig S1‐S4
