# Supplementary material for: Detection and isolation of airborne SARS‐CoV‐2 in a hospital setting
Source: Indoor Air. 2022 Mar 27;32(3):e13023. doi: 10.1111/ina.13023 (PMC9111425; doi:10.1111/ina.13023)
Supplement: Supplementary file 1 — Fig S1‐S4 [file INA-32-0-s001.docx]

**Supplementary Figure 1. Illustration of sampling locations in patient and adjoining anteroom.** General layout of patient room with adjoining anteroom. Approximate sampling locations marked (**A**). Room not to scale. THOR on tripod (**B**). THOR dimensions are: 13 cm (L) x13 cm (W) x 7.5 (H). Bed rail (**C**). Ceiling air exhaust vent (**D**). White bar approximates 16 cm.

**Supplementary Figure 2. Comparison of SARS-CoV-2 RNA detection in environmental samples with patient sample.** Ct values for amplification of SARS-CoV-2 by hid-RT-PCR from air and surface samples from the patient room were plotted against Ct values from patient samples (combined naso- and oropharynx swabs) taken at the time of symptom development, also determined by hid-RT-PCR for the virus. Each circle represents an individual sample.

**Supplementary Figure 3. Comparison of hid-RT-PCR against extraction RT-qPCR for SARS-CoV-2 RNA detection in environmental samples.** A set of air and surface samples were analyzed by hid-RT-PCR (circles) and compared to a conventional RNA extraction-based RT-qPCR (squares). Samples are grouped to show cases where sample Ct decreased; turned positive; or increased after RNA extraction.

**Supplementary Figure 4. Electrostatic inactivation of SARS-CoV-2.** A stock solution of SARS-CoV-2 (1x10^6^ PFU/mL) was aliquoted into 4 mL of PBS in a metal dish collector connected to THOR. THOR was then activated (THOR *on*) or left inactive (THOR *off*) for 15 min to expose the virus to the electrostatic field produced by THOR during active sampling. Material from the collector was plated on Vero E6 cells for quantification of SARS-CoV-2 PFUs. PFU data was Log_10_-transformed and an unpaired two-tailed t test used to determine statistical significance. Bars indicate SEM. Dots indicate individual replicates. * Difference between THOR *on* and *off* is statistically significant, p=0.0001.
